# Synthesis, Characterisation and Anti-Fungal Activities of Some New Copper(II) Complexes of Octamethyl Tetraaza-Cyclotetradecadiene

**DOI:** 10.1155/MBD.1999.345

**Published:** 1999

**Authors:** Tapashi G. Roy, Saroj K. S. Hazari, Benu K. Dey, Samar Chakraborti, Edward R. T. Tiekink

**Affiliations:** 1 Department of Chemistry University of Chittagong Chittagong 4331 Bangladesh; 2 Department of Chemistry The University of Adelaide Adelaide 5005 Australia

## Abstract

The ligand Me_8_[14]diene, L, in its free state as well as in the dihydroperchlorate form, L.2HClO_4_, coordinates copper(ll) in different salts to yield a series of [CuLX_x_] X_y_(H_2_O)_z_ complexes where X = NO_3_, ClO_4_, NCS, Cl and Br; x and y may have values of 0 or 2 and z = 0, 1 or 2. The complex, [CuL(ClO_4_)_2_].2H_2_O is found to undergo axial ligand substitution reactions with SCN^-^, NO_3_ and Cl^-^ to give a variety of substitution derivatives: [CuL(ClO_4_)_m_ X_n_] where X = NCS, NO_3_ and Cl; m = 0 or 1, and n = 1 or 2. The complexes .have been characterised on the basis of analytical, spectroscopic, magnetic and conductance data. The anti-fungal activities of the ligand and its complexes have been investigated against a range of phytopathogenic fungi.


### SYNTHESIS, CHARACTERISATION AND ANTI-FUNGAL ACTIVITIES OF SOME NEW COPPER(II) COMPLEXES OF OCTAMETHYL TETRAAZA-CYCLOTETRADECADIENE

Tapashi G. Roy*[1], Saroj K. S. Hazari[1], Benu K. Dey[1], Samar Chakraborti[1] and Edward R. T. Tiekink*[2]

[1] Department of Chemistry, University of Chittagong, Chittagong - 4331, Bangladesh
[2] Department of Chemistry, The University of Adelaide, Australia 5005

**Abstract**

The ligand $Me_8$[14]diene, L, in its free state as well as in the dihydroperchlorate form, $L.2HClO_4$, coordinates copper(II) in different salts to yield a series of $[CuLX_x]X_y.(H_2O)_z$ complexes where X = $NO_3$, $ClO_4$, NCS, Cl and Br; x and y may have values of 0 or 2 and z = 0, 1 or 2. The complex, $[CuL(ClO_4)_2].2H_2O$ is found to undergo axial ligand substitution reactions with $SCN^-$, $NO_3^-$ and $Cl^-$ to give a variety of substitution derivatives: $[CuL(ClO_4)_mX_n]$ where X = NCS, $NO_3$ and Cl; m = 0 or 1, and n = 1 or 2. The complexes have been characterised on the basis of analytical, spectroscopic, magnetic and conductance data. The anti-fungal activities of the ligand and its complexes have been investigated against a range of phytopathogenic fungi.

### 1. Introduction

The synthetic macrocycles and their complexes have attracted attention owing to their wide variety of applications. This contribution focuses on the synthesis and characterisation of a series of copper(II) complexes of octamethyl tetraazacyclotetradecadiene with the view of investigating their anti-fungal activities. It has been established that stereospecific condensation of 1,2-diaminopropane with acetone yields only the 3,10-C-meso isomer of the macrocycle 3,5,7,7,10,12,14,14-octamethyl-1,4,8,11-tetraazacyclotetradeca-4,11-diene, L, as determined by $^1$H NMR [1,2] and X-ray crystallography [3].

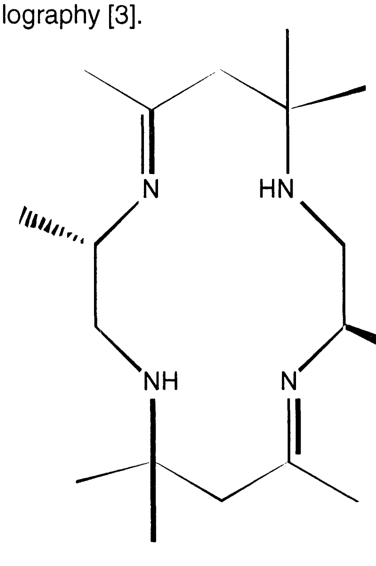

Template synthesis of four-coordinate square planar copper(II) complexes of L has been achieved [1], but five- or six-coordinate copper(II) complexes of this ligand have not been reported thus far. Owing to the steric hindrance of eight methyl groups in L, it was expected that the preparation of five- or six-coordinate complexes containing this ligand would be difficult. In one study, Bembi and coworkers [4] synthesised a number of six-coordinate cobalt(III) complexes $[CoL^xCl_2](ClO_4)$, where $L^x$ = isomeric $Me_8$[14]anes; N-chiral isomers were separated and characterised. In another study [5], the preparation of six-coordinate dichlorocobalt(III) complexes of L was achieved. Subsequently, in an recent study, Hazari *et al.* [6] were successful in preparing some six-coordinate copper(II) complexes containing the saturated isomeric $Me_8$[14]anes analogues of L, $[Cu(Me_8[14]anes)X_2]^{n+}$ (where X = $NO_3$, $H_2O$, Cl and Br; n = 0, 1 or 2). Hence, it seemed likely that higher coordination number copper(II) complexes could also be prepared with L. In this context, a number of four- and six-coordinate copper(II) complexes have been isolated and their anti-fungal activities, as well as those of L, investigated.

### 2. Experimental

### 2. 1 Synthesis

The dihydroperchlorate salt of the ligand, 3,10-C-meso-$Me_8$[14]diene.$2HClO_4$, hereafter L.$2HClO_4$, was synthesised according to the literature method [1]. The free ligand, 3,10-C-meso-$Me_8$[14]diene, L, was obtained by extracting the ligand with chloroform from a suspension of L.$2HClO_4$ in water at a pH above 12; m. pt 117°C. Found N, 18.15 %. $C_{18}H_{36}N_4$ requires N, 18.18 %.

#### *2.1.1. Copper(II) diperchlorato complex, $[CuL(ClO_4)_2].2H_2O$*

L.$2HClO_4$ (0.51g, 1.0 mmol) and $CuCl_2.2H_2O$ (0.171 g, 1.0 mmol) were dissolved separately in hot dry EtOH (20 ml) and mixed while hot. A blue colour appeared immediately. The reaction mixture was heated on a water bath for 30 min during which time the solution changed colour from blue to violet. On heating for a further 30 min, the volume was reduced to *ca* 5 ml. After cooling to room temperature, the purple product was filtered off, washed with dry EtOH and then with $Et_2O$.

On recrystallisation from absolute EtOH, a reddish-pink product, $[CuL(ClO_4)_2].2H_2O$, was obtained. Found N, 9.20; Cu, 10.43 %. $C_{18}H_{40}Cl_2CuN_4O_{10}$ requires N, 9.23; Cu, 10.47 %. The same product was also obtained by the reaction of L.$2HClO_4$ with $Cu(ClO_4)_2.6H_2O$ as well as with $Cu(NO_3)_2.3H_2O$.

#### *2. 1. 2. Copper(II) chloroperchlorato complex, $[CuLCl(ClO_4)]$*

$[CuL(ClO_4)_2].2H_2O$ (0.61 g, 1.0 mmol) was dissolved in hot MeOH and KCl (0.15 g, 2.0 mmol) was added to this solution. The pink colour of the solution rapidly changed to violet. The mixture was evaporated to dryness on a steam bath and the crude, dried product was extracted with $CHCl_3$. The $CHCl_3$ extract was taken to dryness on a steam bath to yield a violet product, $[CuLCl(ClO_4)]$. Found N, 11.06; Cu, 12.54 %. $C_{18}H_{36}Cl_2CuN_4O_4$ requires N, 11.05; Cu, 12.53 %.

#### *2.1.3. Copper(II) nitratoperchlorato complex, $[CuL(NO_3)(ClO_4)]$*

$[CuL(ClO_4)_2].2H_2O$ (0.61 g, 1.0 mmol) was dissolved in hot dry MeOH (30 ml) and $KNO_3$ (0.20 g, 2.0 mmol) was added to it, while hot. The solution changed colour from light pink to deep pink immediately. After heating for *ca* 10 min the colour rapidly changed to reddish-pink. Heating was continued for further 15 min and filtered to remove insoluble $KClO_4$. The filtrate was then evaporated to dryness. The crude dried product was redissolved in a minimum quantity of $CHCl_3$ and then again filtered to remove white residue from the solution. The filtrate was again evaporated to dryness on a steam bath to result a pink product, $[CuL(NO_3)(ClO_4)]$. Found N, 13.08; Cu, 11.92 %. $C_{18}H_{36}ClCuN_5O_7$ requires N, 13.13; Cu, 11.91 %.

#### *2.1.4. Copper(II) diisothiocyanato complex, $[CuL(SCN)_2]$*

$[CuL(ClO_4)_2].2H_2O$ (0.61 g, 1.0 mmol) and KCNS (0.19 g, 2.0 mmol) were dissolved separately in hot dry MeOH (5 ml) and mixed while hot. The initial pink colour rapidly changed to violet. On heating the solution for *ca* 5 min, the mixture was filtered to remove insoluble $KClO_4$ produced in the reaction. The filtrate was completely dried on a steam bath and the dried product was extracted with $CHCl_3$. The $CHCl_3$ extract was concentrated on a steam bath to a volume of *ca* 5 ml until precipitation commenced. After cooling to room temperature, the violet product was filtered off, washed with dry EtOH followed by $Et_2O$ and finally dried *in vacuo*; m. pt > 280°C. Found N, 17.17; S, 13.10; Cu, 13.00 %. $C_{20}H_{36}CuN_6S_2$ requires N, 17.21; S, 13.13; Cu, 13.01 %.

**Table 1.** Selected IR bands (KBr optics, $cm^{-1}$)

| Complex | ν(NH) | ν(C=N) | ν(CH) | $\nu(CH_3)$ | ν(CC) | ν(MN) | ν(OH) | δ(HOH) | Other bands |
|---|---|---|---|---|---|---|---|---|---|
| $[CuL(ClO_4)_2].2H_2O$ | 3200m | 1650s | 2965m | 1380m | 1135sh | 570w | 3420s | 1650s | 1130, 1080s, 915vw,620s, $\nu(ClO_4)$ |
| $[CuLCl(ClO_4)]$ | 3240vw | 1650m | 2980vw | 1390vw | 1125m | 570 | - | - | 1125m, 1075w, 940vw, 625m, $\nu(ClO_4)$, 265m ν(MCl) |
| $[CuL(NO_3)(ClO_4)]$ | 3240vw | 1650m | 2960sh | 1380s | 1125m | 575m | - | - | 1125s, 1080m, 940w, 620s, $\nu(ClO_4)$ 1410sh, 1300m, $\nu(NO_3)$, 320m, νMO |
| $[CuL(NCS)_2]$ | 3260w | 1650s | 2965w | 1375m | 1130m | 570m | - | - | 2025s, νCN; 820m, νCS; 470w, ν(NCS) |
| $[CuL](NO_3)_2$ | 3260w | 1645m | 2960w | 1375m | 1140w | 575m | - | - | 1375s $\nu(NO_3)$ |
| $[CuL(NO_3)_2].H_2O$ | 3240w | 1625s | 2960w | 1380s | 1130w | 575m | 3440w | 1625m | 1435m ,1325sh, $\nu(NO_3)$; 250 m, νMO |
| $[CuLCl_2].H_2O$ | 3135m | 1650m | 2970s | 1370s | 1160s | 570w | 3320w | 1650m | 275m, νMCl |
| $[CuL]Br_2.2H_2O$ | 3180w | 1625w | 2970s | 1380s | 1180m | 535s | 3375s | 1625s | - |
| $[CuLBr_2].2H_2O$ | 3175m | 1620w | 2970m | 1380m | 1180s | 535m | 3375m | 1620m | 240m, νMBr |

*2.1.5. Copper(II) nitrate complexes, $[CuL](NO_3)_2$ and $[CuL(NO_3)_2].H_2O$*

L (0.31g, 1.0 mmol) and $Cu(NO_3)_2.3H_2O$ (0.19 g, 1.0 mmol) were dissolved separately in hot MeOH (25ml) and mixed while hot. A violet colour appeared immediately. The reaction mixture was heated on a water bath for *ca* 20 min during which time a green precipitate settled at the bottom of the container. After cooling to room temperature, the green complex, $[CuL](NO_3)_2$, was filtered off, washed with dry EtOH followed by $Et_2O$ and dried *in vacuo*; m. pt 135°C. Found N, 16.90; Cu, 12.80 %. $C_{18}H_{36}CuN_6O_6$ requires N, 16.94; Cu, 12.81 %.

After separating the green product, the mother liquor was concentrated to *ca* 5ml. On cooling, a pink product precipitated out. After 30 min., the product, $[CuL(NO_3)_2].H_2O$ was filtered off, washed and dried in the same manner as above; dec. pt 240°C. Found N, 16.30; Cu, 12.33 %. $C_{18}H_{38}CuN_6O_7$ requires N, 16.35; Cu, 12.36 %.

*2.1.6. Copper(II) dichloro complex, $[CuLCl_2].H_2O$*

L (0.31g, 1.0 mmol) and $CuCl_2.2H_2O$ (0.14 g, 1.0 mmol) were dissolved in hot MeOH (30 ml). A violet colour appeared immediately. On heating the colour changed to light red. The solution was reduced to *ca* 10 ml on a steam bath and on cooling a red product, $[CuLCl_2].H_2O$, precipitated out which was filtered, washed with absolute EtOH and finally dried *in vacuo*; m. pt > 280°C. Found N, 12.19; Cu, 13.77 %. $C_{18}H_{38}Cl_2CuN_4O$ requires N, 12.16; Cu, 13.79 %.

*2.1.7. Copper(II) bromide complexes, $[CuL]Br_2.2H_2O$ and $[CuLBr_2].2H_2O$*

L (0.318g, 1.0 mmol) and $CuBr_2$ (0.2238 g,1.0 mmol) were mixed together in hot dry MeOH (40 ml). A red colour appeared immediately. On heating the solution for *ca* 5 min on a water bath, a green complex was observed. This was separated by filtration; dec. pt 140°C. Due to insufficient quantity, further analysis could not be performed.

After separating the green product, the mother liquor was further heated for 30 min during which time the solution turned a violet colour and finally evaporated. The dry product was extracted with $CHCl_3$. The $CHCl_3$ extract was then evaporated to dryness to yield a violet product, $[CuLBr_2].2H_2O$; dec. pt 240°C. Found N, 9.81; Cu, 11.17 %. $C_{18}H_{40}CuBr_2N_4O_2$ requires N, 9.87; Cu, 11.19 %.

The above violet product, on exposure to air at room temperature, produced a pink product, $[CuL]Br_2.2H_2O$; dec. pt 240°C. Found N, 9.82; Cu, 11.17 %. $C_{18}H_{40}Br_2CuN_4O_2$ requires N, 9.87; Cu, 11.19 %.

The pink product on heating to 70 - 80°C reverts to the violet product.

### 2. 2 Physical measurements

Visible spectra were recorded on a Shimadzu UV-visible spectrophotometer. The mass spectrum was measured at the Department of Radiochemistry and Biophysics, Nigata College of Pharmacy, Nigata, Japan. Conductance measurements were carried out on a conductivity bridge model Hanna Instruments HI-8820 at 25 ± 0.1°C. Magnetic measurements were made on Gouy Balance which was calibrated using $Hg[Co(NCS)_4]$. IR spectra were recorded on a Perkin-Elmer model-883 infrared spectrophotometer as KBr disks.

### 2. 3 Elemental analysis

For the analysis of nitrogen, Kjeldahl's method, for copper standard titrimetric methods, and for sulphur standard gravimetric methods have been employed.

### 2.4. Anti-fungal activities

The actifungal activities of $L.2HClO_4$, and its copper complexes (*in vitro*) against some selected phytopathogenic fungi were assessed by the poisoned food technique. Potato Dextrose Agar (PDA) was used as a growth medium. DMF was used as the solvent, initially to prepare solutions of the compounds. The solutions were then mixed with the sterilised PDA to maintain the concentration of the compounds at 0.01 %; 20 ml of these were each poured into a petri dish. After the medium had solidified, a 5 mm mycelial disc for each fungus was placed in the centre of each

**Table 2.** Physical appearance, electronic spectral,[a] magnetic and conductivity data

| Complex | Colour in solid state | Solvent | d-d bands $\lambda_{max}$(nm) (log $\varepsilon_{max}$) | Colour in DMF | Molar conductivity $ohm^{-1}cm^2 mol^{-1}$ | Colour in $CHCl_3$ | $ohm^{-1}cm^2 mol^{-1}$ | Colour in water | $ohm^{-1}cm^2 mol^{-1}$ | Magnetic moment $\mu_{eff}$ B.M. |
|---|---|---|---|---|---|---|---|---|---|---|
| $[CuL(ClO_4)_2].2H_2O$ | red-pink | Nujol<br>chloroform<br>DMF<br>Water | 495<br>495(1.61)<br>532(2.13)<br>516(2.15) | pink-violet | 150 | red-pink | 0.0 | pink | 180 | 1.91 |
| $[CuLCl(ClO_4)]$ | violet | DMF | 575(2.01) | pink-violet | 50 | violet | 0.0 | pink | 190 | 1.75 |
| $[CuL(NO_3)(ClO_4)]$ | red-pink | Nujol<br>DMF | 500<br>525(1.92) | bright-pink | 94 | red-pink | 0.0 | pink | 190 | 1.89 |
| $[CuL(NCS)_2]$ | violet | DMF | 537(2.16) | blue-violet | 110 | violet | 0.0 | pink | 150 | 1.82 |
| $[CuL(NO_3)_2].H_2O$ | violet | DMF<br>chloroform | 524(2.08)<br>509(1.66) | blue-violet | 124 | violet | 0.0 | pink | 180 | 1.74 |
| $[CuLCl_2].H_2O$ | red | DMF | 551(2.00) | red | 12 | - | - | - | - | 1.75 |
| $[CuL]Br_2.2H_2O$ | bright-pink | DMF | 516(1.78) | pink-violet | 64 | violet | 0.0 | - | - | 1.83 |
| $[CuLBr_2].2H_2O$ | violet | DMF<br>chloroform | 565(2.10)<br>567(2.19) | pink-violet | 73 | violet | 0.0 | - | - | 1.90 |

a $\varepsilon_{max}$ = maximum molar extinction coefficient in $dm^3 mol^{-1} cm^{-1}$

assay plate against the control. Linear growth of the fungus was measured in mm after five days of incubation at 25 ± 2$^0$C.

### 3. Results and discussion

The mass spectrum of free ligand L gave m/z 308 corresponding to the molecular ion. On the basis of $^1$H NMR [1,2] and X-ray crystallography [3] L has been assigned the structure as shown in the *Introduction*. On reaction with copper(II) salts, L.2HClO$_4$ and L yield both four- and six-coordinate complexes of the general formula $[CuLX_x]X_y(H_2O)_z$, where X = NO$_3$, ClO$_4$, NCS, Cl and Br; x and y may have values of 0 or 2; z = 0, 1 or 2. The complex $[CuL(ClO_4)_2].2H_2O$ undergoes substitution reactions to give products $[CuL(ClO_4)_mX_n]$ where X = SCN, NO$_3$ and Cl; m = 0 or 1; n = 1 or 2. Since the $^1$H NMR could not be measured for these paramagnetic complexes, exact stereochemistries could not be determined except for that of $[CuL(ClO_4)_2].2H_2O$ for which a single crystal structure analysis has been undertaken and reported elsewhere [7]. It is assumed that the stereochemistries of the substitution products remain the same as the parent compound. Characterisation of the complexes could be achieved by IR and UV/VIS spectroscopic data as well as by magnetochemical and conductance measurements. Physical and spectroscopic data are presented in Tables 1 and 2.

An examination of molecular models show that owing to the presence of two chiral N-centres in L, up to four diastereoisomeric complexes of the same geometry may result. Out of these possibilities, all are not stable in solid state. In this study, only one diastereoisomer of each complex was isolated.

### *3.1 Copper(II) diperchlorato complex*

The interaction of copper(II) chloride with L.2HClO$_4$ yields a reddish pink product, $[CuL(ClO_4)_2].2H_2O$. The same product could also be prepared by the reaction of L.2HClO$_4$ with $Cu(ClO_4)_2.6H_2O$ as well as with $Cu(NO_3)_2.3H_2O$ as demonstrated by infrared spectroscopy.

The IR spectrum exhibits bands at 1130, 1080, 915 and 620 cm$^{-1}$ due to the perchlorate group. The splitting of a band at 1100 cm$^{-1}$ into 1130 cm$^{-1}$ and 1080 cm$^{-1}$ is an indication of presence of coordinated perchlorate and the position of the bands strongly supports a unidentate mode of coordination [8]. Presence of a $\nu_{OH}$ band at 3420 cm$^{-1}$ and a $\delta_{HOH}$ band at 1650 cm$^{-1}$ overlapping with a $\nu_{CN}$ band are attributed to the presence of lattice water [9]. The spectrum further shows the appearance of bands due to C=N, NH, C-C, CH$_3$ in the expected regions. Selected IR bands for all complexes are collected in Table 1. The conductance value at 0.0 ohm$^{-1}$ cm$^2$ mol$^{-1}$ (Table 2) for this complex in CHCl$_3$ solution is an indication of the non-electrolytic nature of the complex, *i.e.* both ClO$_4$ groups are in the coordination sphere. This assignment, *i.e.* an octahedral geometry, has been confirmed by an X-ray structure determination [7]. However, in DMF and water where the colour is changed to pink-violet and pink, respectively, the conductance values corresponding to 1:2 electrolytes indicate that solvent molecules replace perchlorate in the coordination sphere as has been noted in related systems [6]. It is possible that in case of DMF, being relatively large molecule, two molecules may not enter the coordination sphere but rather they may force the anions out of the sphere to form a square planar complex, $[CuL](ClO_4)_2$ as has been observed in analogous complexes [10].

It has been shown that copper(II) centres in macrocycles generally have square planar or tetragonally distorted octahedral geometries and that these give rise to broad bands in the visible region due to overlap of $A_{1g} \rightarrow B_{1g}$, $B_{2g} \rightarrow B_{1g}$ and $E_g \rightarrow B_{1g}$ transitions [10]. The $[CuL(ClO_4)_2].2H_2O$ complex shows a broad *d-d* band at 495 nm in the solid state and in CHCl$_3$ solution, at 532 nm in DMF and 516 nm in water (Table 2) consistent with the above. The magnetic moment 1.91 B.M. is in good agreement with the copper(II) complex having one unpaired electron.

### *3. 2 Copper(II) monochloroperchlorato complex*

When $[CuL(ClO_4)_2].2H_2O$ was allowed to react with KCl in methanol solution, a violet complex, $[CuLCl(ClO_4)]$, was produced which was purified by extraction with chloroform.

The IR spectrum of the complex reveals bands at 1125, 1075, 940 and 625 $cm^{-1}$ due to coordinated unidentate $ClO_4$. A band at 265 $cm^{-1}$ can be assigned to the Cu-Cl stretching frequency. The conductance at 0.0 $ohm^{-1}$ $cm^2$ $mol^{-1}$ in violet $CHCl_3$ solution fully supports the above assignment. However, the molar conductivity value in pink-violet DMF solution corresponding to a 1:1 electrolyte may be accounted for by the replacement of weakly bound $ClO_4$ by DMF or by the presence of an equilibrium mixture of octahedral $[CuLCl(ClO_4)]$ and square planar $[CuL]Cl(ClO_4)$. Further, the conductance value (190 $ohm^{-1}$ $cm^2$ $mol^{-1}$) of pink aqueous solution corresponding to a 1:2 electrolyte clearly suggests that the anions are replaced by $H_2O$ molecules [6] as described earlier. The magnetic moment and electronic data are consistent with an originally tetragonally distorted octahedral structure. It has been concluded that once the octahedral complex is formed, substitution of the axial ligands takes place without change of conformation as established in the case of cobalt(III) complexes containing the same ligand [11].

### *3. 3 Copper(II) mononitratoperchlorato complex*

Reaction of $[CuL(ClO_4)_2].2H_2O$ with $KNO_3$ in methanol solution produced a mixture of pink and white product. On extraction with chloroform and evaporation of the solvent, pure reddish-pink $[CuL(NO_3)(ClO_4)]$ was isolated. The remaining white material was not characterised.

In the IR spectrum of the complex, a band at 1380 $cm^{-1}$, similar to that found in the spectrum of free ligand can be assigned to absorptions due to $CH_3$ groups [12]. A medium band at 1300 $cm^{-1}$ and a shoulder at 1410 $cm^{-1}$, that overlapped with the band due to methyl groups, are attributed to coordinated nitrate group. The separation of these bands by 110 $cm^{-1}$ and the appearance of a single sharp M-O band at 320 $cm^{-1}$ is indicative of unidentate nitrate [13]. The spectrum further shows the bands at 1125, 1080, 940 and 620 $cm^{-1}$ which can be safely assigned to the presence of a unidentate perchlorate group. The conductance value (0.0 $ohm^{-1}$ $cm^2$ $mol^{-1}$) of a reddish-pink $CHCl_3$ solution of this complex is indicative of the non-electrolytic nature of the complex, *i.e.* nitrate and perchlorate are in the coordination sphere. However, the molar conductivity (94 $ohm^{-1}$ $cm^2$ $mol^{-1}$) in DMF corresponds to a 1:1 electrolyte and is attributed to an equilibrium between octahedral $[CuL(NO_3)(ClO_4)]$ and square planar $[CuL](NO_3)(ClO_4)$ [10]. The electronic spectral and magnetic data, Table 2, are consistent with the tetragonally distorted octahedral structure.

### *3.4 Copper(II) diisothiocyanato complex*

$[CuL(ClO_4)_2].2H_2O$ was reacted with KSCN in the ratio of 1:2 in methanol solution to produce a mixture of white and violet products. This mixture was extracted with chloroform which on evaporation yielded a violet product, characterised as $[CuL(NCS)_2]$.

In the IR spectrum of $[CuL(NCS)_2]$, the appearance of distinct, sharp $\nu_{CN}$ at 2025 $cm^{-1}$, $\nu_{CS}$ at 820 $cm^{-1}$ and $\nu_{NCS}$at 470 $cm^{-1}$ bands is a good indication of the coordination of NCS ions and their positions fully supports the N bonded thiocyanate group [13]. This assignment is in good agreement with the fact that generally first row transition metal complexes of thiocyanate form M-N bonds [13]. Absence of bands at around 1100 $cm^{-1}$, 920 $cm^{-1}$ and 625 $cm^{-1}$ in this complex reveals that although this complex has been prepared from a diperchlorato precursor, the perchlorate ions are fully replaced by thiocyanate ions.

The molar conductivity value of 0.00 $ohm^{-1}$ $cm^2$ $mol^{-1}$ of this complex in chloroform solution strongly supports the non-electrolytic nature of the complex, *i.e.* both thiocyanate ions are in the coordination sphere. The conductance value in DMF solution, corresponding to an 1:1 electrolyte, can be explained in terms of the phenomena discussed above (Section 3.3).

### *3.5 Copper(II) nitrate complexes*

The interaction of free ligand L with copper(II) nitrate in the ratio of 1:2 in methanol solution yielded one green fraction immediately and a violet product later. The green product was found to be insoluble in almost all solvents and the quantity isolated was insufficient for full characterisation. The IR spectrum of the green product reveals a sharp, intense band at 1375 $cm^{-1}$ that may be attributed to ionic, non-coordinating $NO_3$ and methyl groups [12]. The spectrum further shows all the characteristic bands as observed for the free ligand. From these data it is proposed that the complex may have a square planar geometry of molecular formula $[CuL](NO_3)_2$.

The IR spectrum of violet $[CuL(NO_3)_2].H_2O$ exhibits a band at 1380 $cm^{-1}$ which may be due to the $CH_3$ groups. Presence of two bands at 1435 $cm^{-1}$ and 1325 $cm^{-1}$ are attributed to nitrate groups. The separation of the bands by 110 $cm^{-1}$ and a single M-O stretching band at 250 $cm^{-1}$ are indications of coordinated unidentate nitrate groups [13]. The presence of lattice water is indicated by the presence of bands at 3440 $cm^{-1}$ and 1625 $cm^{-1}$ [9]. The conductance value of 0.0 $ohm^{-1}$ $cm^2$ $mol^{-1}$ in chloroform solution shows that the complex is essentially a non-electrolyte. However, in DMF and water, the molar conductivity values indicate that this complex behaves as an 1:2 electrolyte which may be due to the formation of square planar $[CuL](NO_3)_2$ and octahedral $[CuL(O_2H)_2](NO_3)_2$ complexes, respectively. Based on the above evidence, a distorted octahedral geometry is proposed for $[CuL(NO_3)_2].H_2O$.

*3. 6. Copper(II) dichloro complex*

Reaction of copper(II) chloride with L in methanol solution yielded red $[CuLCl_2].H_2O$ during the course of the synthesis. A violet solution was found to give this red product which suggested that a different diastereoisomer or geometrical isomer was abundant in solution but only the red product was stable in the solid state. A similar phenomenon was observed in the related copper(II) / chloro complexes containing the isomeric $Me_8[14]$anes, $L^x$ [6].

The IR spectrum of the red complex reveals $\nu_{OH}$ band at 3300 $cm^{-1}$ and a $\delta_{HOH}$ band at 1650 $cm^{-1}$, which is an indication of the presence of lattice water in this complex. The appearance of a band at 275 $cm^{-1}$ may be assigned to a Cu-Cl stretching frequency.

The molar conductivity at 12 $ohm^{-1}$ $cm^2$ $mol^{-1}$ in DMF solution shows that the complex is essentially a non-electrolyte. Thus, an irregular octahedral geometry is proposed for $[CuLCl_2].H_2O$.

*3. 7. Copper(II) bromide complexes*

The interaction of L with copper(II) bromide in methanol solution produced green $[CuL]Br_2$, pink $[CuL]Br_2.2H_2O$ and violet $[CuLBr_2].2H_2O$. The green product was isolated in insufficient quantity to be characterised fully. The same type of green product was also obtained during the reaction of L with copper(II) nitrate for which a square planar geometry was proposed. Both of these green products are expected to be the same diastereoisomer. The pink product was obtained at room temperature and the violet product can be isolated in the absence of moisture or by heating the pink product to 70 - 80°C. Moreover, the violet product reverts to the pink one on exposure to moisture.

The IR spectrum of pink $[CuL]Br_2.2H_2O$ reveals sharp $\nu_{OH}$ and $\delta_{HOH}$ bands indicating lattice water; absence of a M-O band around 450 $cm^{-1}$, assigned for aquo complexes, demonstrates that water is not coordinated in this complex. Further, no band is seen around 250 $cm^{-1}$ indicating that the bromide is non-coordinating. Since copper(II) macrocyclic complexes can have square planar or tetragonally distorted octahedral geometries and neither $Br^-$ nor $H_2O$ are coordinated, a square planar geometry is most likely.

The *d-d* band at 516 nm in the electronic spectrum of $[CuL]Br_2.2H_2O$ is consistent with the above assignment. This product was found to change its colour in almost all solvents. So, the expected conductance value for an 1:2 electrolyte could not be obtained in any solvent. Moreover, the conductance value (64 $ohm^{-1}$ $cm^2$ $mol^{-1}$) of a pink-violet DMF solution, corresponding to an 1:1 electrolyte, is accounted for by the equilibrium between $[CuL]Br_2.2H_2O$ and $[CuLBr_2].2H_2O$. This observation suggests that the non-coordinating $Br^-$ ions of $[CuL]Br_2.2H_2O$ are forced into the coordination sphere to some extent. A similar observation was found for analogous copper(II) / bromo complexes of $L^x$ [6]. On the basis of above analysis, a square planar structure is predicted for $[CuL]Br_2.2H_2O$.

The IR spectrum of violet $[CuLBr_2].2H_2O$ shows a similar pattern to that found for $[CuL]Br_2.2H_2O$ except for the appearance of an additional band at 240 $cm^{-1}$ that is assigned to a Cu-Br stretching frequency. The non-electrolytic nature of this complex in $CHCl_3$ solution strongly supports a tetragonally distorted octahedral complex.

### 3. 8. Synthetic Overview

Stable complexes of the general formula $[CuLX_x]X_y.(H_2O)_z$ (X = $NO_3$, $ClO_4$, NCS, Cl and Br; x and y may have values of 0 or 2 and z = 0, 1 or 2) and substitution products of $[CuL(ClO_4)_2].2H_2O$ with the general formula $[CuL(ClO_4)_mX_n]$ (X = NCS, $NO_3$ and Cl; m = 0 or 1 and n = 1 or 2) have been isolated. Some of these were found to be inter-convertible by the rearrangement of the ligand donor set. The conductance of all complexes determined in aqueous solution indicated the presence of 1:2 electrolytes. This behaviour may be accounted for by the formation of diaquo, octahedral $[CuL(H_2O)_2]^{2+}$ cations or square planar $[CuL]^{2+}$ cations. Structure assignment, in terms of coordination of the anions was achieved primarily on the basis of IR spectroscopy. Magnetic moments (Table 2) indicate normal behaviour for these $d^9$ systems. This study demonstrates that it is possible to form tetragonally distorted octahedral copper(II) complexes with the sterically congested macrocycle with eight peripheral methyl groups, L. Since the two methyl groups of the chiral carbon atoms and the two methyl groups at C=N are equatorially orientated in L, the participation of weakly-coordinating, relatively large ions such as perchlorate in the coordination sphere is allowed. Small and labile ligands such as $Br^-$ was found to have facile entrance and exit in the coordination sphere.

### 3. 9. Fungitoxicity study

The anti-fungal activities of the ligand and some of its complexes are summarised in Table 3.

**Table 3.** *In-vitro* anti-fungal activities of $L.2HClO_4$ and some copper(II) complexes

| Compounds | % inhibition of mycelial growth | | |
|---|---|---|---|
| | *Alternaria alternata* | *Carvularia lunata* | *Macrophomina phaseolina* |
| $L.2HClO_2$ | 21.5 | 16.0 | 33.3 |
| $[CuL(ClO_4)_2].2H_2O$ | 15.4 | 12.0 | 17.6 |
| $[CuLCl(ClO_4)]$ | 12.1 | 10.0 | 15.9 |
| $[CuL(NO_3)(ClO_4)]$ | 11.7 | 10.0 | 15.0 |
| $[CuL(NCS)_2]$ | 10.9 | 7.9 | 10.9 |
| $[CuL(NO_3)_2].H_2O$ | 11.0 | 8.2 | 11.0 |
| $[CuLCl_2]. H_2O$ | 13.1 | 9.0 | 10.8 |
| $[CuL]Br_2.2H_2O$ | 11.0 | 8.0 | 10.9 |
| $[CuLBr_2].2H_2O$ | 10.9 | 8.0 | 11.0 |

Screens have been conducted against three selected phytopathogenic fungi: i) *Alternaria alternata,* ii) *Curvularia lunata,* and iii) *Macrophomina phaseolina.* The activities of the free ligand and its complexes against *Macrophomina phaseolina* are generally greater than those against the other two fungi. This is in contrast to that observed in case of its saturated isomeric macrocycles $Me_8$[14]anes and their copper(II) complexes [6], where the activities were greater against *Alternaria alternata* than those against other two fungi. This observation suggests that the diene ligand may have a different effect on these organisms. The activities of this ligand were found to decrease upon coordination to copper(II), as usual.

The fungitoxicities are generally lower than those of related sulphur containing Schiff bases and their complexes [14]. It is noteworthy that the decrease in activity upon coordination of this particular ligand was found to be less compared to that of sulphur containing compounds [14] but comparable to that observed in case of its saturated macrocyclic analogues [6].

**Acknowledgments**

Financial support from Third World Academy of Sciences (TWAS), Trieste, Italy awarded to Dr T.G. Roy is gratefully acknowledged.

**References:**

1. N. F. Curtis, D. A. Swann, T. N. Waters and I. E. Maxwell, *J. Am. Chem. Soc.* **91** (1969) 4588.
2. T. Ito and D. H. Busch, *Inorg. Chem.*, **13** (1974) 1770.
3. D. A. Swann, T. N. Waters and N. F. Curtis, *J. Chem. Soc., Dalton Trans* (1972) 1115.
4. R. Bembi, M. G. B. Drew, R. Singh and T. G. Roy, *Inorg. Chem.*, **30** (1991) 1403.
5. T. H. Hu, B. H. Chen and C. S. Chung, *J. Chin. Chem. Soc. (Taipee)*, **39** (1992) 343.
6. S. K. S. Hazari, T. G. Roy, B. K. Dey, S. C. Das and E. R. T. Tiekink, *Metal-Based Drugs,* **4** (1997) 255.
7. S. K. S. Hazari, T. G. Roy, B. K. Dey, S. Chakraborti and E. R. T. Tiekink, *Z. Kristallogr.-New Crystal Structures,* **214** (1999) 51.
8. K. Nakamoto, Infrared and Raman spectra of inorganic and coordination compounds (1986) John Willey & Sons, 4th edn., New York, NY, USA.
9. R. W. Hay, B. Jeragh, G. Ferguson, B. Kaitner and B. L. Ruhl., *J. Chem. Soc. Dalton Trans*, (1982) 1531.
10. R. Bembi, R. Singh, S. Aftab, T. G. Roy and A. K. Jhanjii, *J. Coord. Chem.*, **14** (1985), 119.
11. S. Chakrabarti, M. Sc. Thesis, University of Chittagong (1998).
12. R. T. Conley, Infrared Spectroscopy (1966), Allen and Beacon Inc., Boston MA, USA, Ch-5.
13. Nakamoto, Infrared spectra of inorganic and coordination compounds, (1963) John Wiley, New York, NY, USA.
14. S. K. S. Hazari, B. K. Dey and B. Genguli, unpublished results.

**Received: August 18, 1999 - Accepted: September 27, 1999 - Received in revised camera-ready format: August 27, 1999**

